# Psychometric evaluation of the Geneva Sentimentality Scale in Chinese college students

**DOI:** 10.1002/pchj.808

**Published:** 2024-10-29

**Authors:** Ting Wu, Nan Nan Wu, Chong Zeng Bi, Yan Wei Yin, Xiao Rong Chen, Tong Yue

**Affiliations:** ^1^ Research Center of Psychology and Social Development, Faculty of Psychology Southwest University Chongqing China; ^2^ Preschool Education Department Chongqing Preschool Education College Chongqing China

**Keywords:** being moved, Chinese college students, emotion, psychometric properties

## Abstract

The Geneva Sentimentality Scale (GSS) measures the experience of being moved and its effects on behavior. Despite the prevalence of this emotional response, it has not been extensively studied in China. This study aims to adapt and revise the GSS for Chinese college students to assess its cross‐cultural consistency. A sample of 1328 students aged 18–24 years participated in the study, with 127 randomly selected for retesting after an 8‐week interval. Exploratory factor analysis reveals that the Chinese version of the GSS includes three factors (emotional labels, tears of joy, and warm feelings in the chest), with a total of nine items. The internal consistency coefficients for the three factors and the overall scale are high, and the total score remains stable over time. Confirmatory factor analysis (CFA) shows that the three‐factor model has a good fit. Multigroup CFA indicates measurement invariance across genders. The results also demonstrate good discriminant and convergent validity for the scale. Overall, the GSS is a reliable and flexible tool for assessing the emotion of being moved among Chinese college students.

## INTRODUCTION

Have you ever reunited with old friends after a long separation or received an unexpected surprise and felt an indescribable emotion that brought tears to your eyes and filled your heart with warmth and love? This unique emotion is known as “being moved” (Seibt et al., 2017; Zickfeld et al., 2020). While people frequently experience this emotion, they often struggle to identify the feeling accurately and may even mistake this particular emotion as a mere by‐product of other positive or negative emotions. Fortunately, recent theoretical and empirical research has started to rectify these misconceptions. Research has shown that the emotion of being moved and the feelings that are subsequently evoked are distinct and significant in various aspects of people's lives. For instance, in the realm of aesthetic appreciation, one study found that being moved mediates the relationship between sad movies and enjoyment (Hanich et al., 2014; Vuoskoski & Eerola, 2017). In interpersonal interactions, being moved has been found to promote altruistic behavior and strengthen social bonds (Blomster Lyshol et al., 2020). Additionally, being moved increases individuals' willingness to engage in collective action (Landmann & Rohmann, 2020; Lizarazo et al., 2022). Importantly, the emotion of being moved can also serve as a beneficial tool for humanity. When issues involve morally or politically relevant behaviors, being moved can act as a powerful persuasive force (Blomster Lyshol et al., 2023; Seibt et al., 2019).

Precisely what kind of emotion is involved in being moved? The kama muta theory, proposed by Zickfeld et al. (2017), provides some insights. Kama muta is a Sanskrit term meaning “being moved by love” and has been suggested as a research term to avoid discrepancies in naming this emotion (Fiske et al., 2019). Kama muta is regarded as a purely positive emotion, which is willingly enjoyed and shared. This specific emotion arises from the sudden intensification of communal bonds, with closeness and morality being key motivators (Seibt et al., 2017). Building on this foundation, Zickfeld, Schubert, Seibt, Blomster, et al. (2019), Zickfeld, Schubert, Seibt, and Fiske (2019) developed the kama muta frequency scale (KAMF), a seven‐item scale designed to measure the frequency and intensity of kama muta experiences. This cross‐cultural study demonstrated that the scale has adequate reliability across different cultural groups (Zickfeld, Schubert, Seibt, Blomster, et al., 2019; Zickfeld, Schubert, Seibt, & Fiske, 2019). However, one must recognize that people can be moved in different ways, for example, interpersonal interactions, the presence of certain positive core values, or the grandeur of natural landscapes (Cullhed, 2020; Landmann et al., 2019). Researchers have argued that being moved is a complex emotion and, depending on the context, often comprises both positive and negative elements (Kuehnast et al., 2014; Menninghaus et al., 2015; Strick & van Soolingen, 2018; Schindler et al., 2022). Therefore, many questions remain about this unique emotion. Current research on kama muta offers only a partial understanding; the extent to which the KAMF scale accurately measures the emotion of being moved is also still a matter of debate.

To avoid controversy and better measure this emotion, Cova et al. (2023) developed the Geneva Sentimentality Scale (GSS). The study viewed being moved as a response to events that are partly or completely positive, which were typically accompanied by pleasant feelings and a warm sensation in the chest (Herting & Schubert, 2022). However, being moved can also include tearful phenomenology, such as tears in the eyes and a lump in the throat (Zickfeld, Schubert, Seibt, Blomster, et al., 2019; Zickfeld, Schubert, Seibt, & Fiske, 2019). In the above study, the GSS demonstrated good measurement validity. The data confirmed that the scale effectively measures the emotion of being moved and exhibits unique strengths among instruments used to measure this emotion. First, the GSS offers a more solid theoretical basis than the KAMF scale; the GSS more adeptly gauges emotional responses to both positive and mixed events. Second, some researchers have used a single‐item scale (“I often feel moved”) to achieve some results. However, this approach is too simplistic to measure the long‐term effects of being moved on an individual's behavior and attitudes (Cova & Boudesseul, 2023). Empirical studies have demonstrated that the GSS measures emotional responses to being moved and predicts the intensity of those responses in both recent and upcoming weeks. More importantly, the GSS has a well‐defined factor structure that aids in assessing the impact of emotional components, scientifically validating the multifaceted nature of being moved. This includes both tears and a warm sensation in the chest (Mori & Iwanaga, 2021). Therefore, the GSS excels with the scale's distinct factor structure and proven reliability, serving as a versatile and thorough measure of emotional responses and their influence on cognition and behavior.

In China, little attention has been given to the definition of “being moved.” However, the concept significantly influences education, as demonstrated by the popularity in school settings of documentaries like “Touching China.” This indicates that, in China, the emotion of being moved holds substantial research value. Additionally, individual emotional responses can vary across cultures, and this raises the question: do complex emotions such as being moved elicit distinctive responses in Chinese culture? Previous research has found that people often report feeling shaky, wanting to cry, and experiencing warmth in the center of their chest when moved (Cova et al., 2023). Do these responses consistently occur within the Chinese population? To explore this issue, this study begins with the measurement instrument, examining whether the three factors of the GSS scale demonstrate good reliability and validity in the Chinese cultural context. Verifying this study's hypothesis would suggest that the emotional reaction of being moved is similar across different cultural contexts. This, in turn, would indicate that being moved is a universal, culturally independent, and stable emotion. Thus, a discussion of this emotion, alongside emotions like happiness and anger, is necessary in a research context. Understanding how being moved affects human life is crucial for improving and managing societies (Murphy & Bastian, 2020).

Given these observations and considering the reliability of the GSS in the United States and the scale's potential contribution to studying “being moved” in Chinese college students (and future cross‐cultural research), this study adapted and revised a Chinese version of the GSS. This study consists of two parts. First, using item analysis and exploratory factor analysis (EFA), an attempt is made to assess the three‐factor structure of the GSS among Chinese college students. Second, the reliability, validity, and structural stability of the Chinese GSS are tested through reliability and validity analyses and confirmatory factor analysis (CFA). This study hypothesizes that the Chinese GSS is a valid instrument with a three‐factor structure, good reliability, and good validity. Previous studies have shown that individuals who feel moved tend to engage in behaviors similar to gratitude; these are types of prosocial behaviors. Furthermore, Zickfeld, Schubert, Seibt, Blomster, et al. (2019), Zickfeld, Schubert, Seibt, and Fiske (2019) demonstrated that kama muta is highly correlated with empathy. Therefore, this study uses the gratitude scale, the prosocial tendencies measure, and the empathic concern dimension of the interpersonal reactivity index to test the concurrent validity of the Chinese GSS. Additionally, to reinforce the study's validity and affirm that the emotion of being moved is universal, transcending cultural, and gender differences, the GSS scale's consistency across genders is also assessed.

## METHOD

### Participants

Sample 1: Through the distribution of 1400 questionnaires, university students across four Chinese regions were surveyed. After invalid questionnaires were removed, 1328 valid responses were analyzed, giving a 94.86% validity rate. The sample includes 418 males and 910 females, aged 18–24, and various academic years are represented. Using Cheng et al.'s (2013) protocol and SPSS 19.0, the sample is evenly divided into two groups for further analysis. Specifically, Sample 1A is used for item and EFA, and Sample 1B is for CFA and reliability testing.

Sample 2: After 8 weeks, 127 students from Sample 1 were randomly recruited to finish the Chinese GSS. This sample was mainly used to retest reliability.

This project was supervised and approved by the Institutional Review Board of the Faculty of Psychology, Southwest University. Written informed consent was obtained from the participants prior to their participation in the research.

### Measures and procedure

The GSS was translated using Brislin's approach (Brislin, 1986), with initial translations made by bilingual experts, followed by back‐translation for verification. Discrepancies were resolved by translator students, and the final Chinese version was vetted by psychology graduate students for clarity and precision.

Convenience sampling was used to obtain Sample 1, in which all subjects were asked to complete the Chinese GSS, the gratitude scale, the tendency to prosocial behavior scale, and the interpersonal response pointer scale.

#### Being moved

The GSS, finalized by Cova et al. (2023) and consisting of 10 entries, was used to measure being moved. The 10 entries measure subjects' tearfulness and perception of inner warmth when moved. In addition, three entries specifically related to emotional labeling (e.g., “I often have a feeling of being moved”) were used to differentiate moving from other positive emotions. With the exception of tearfulness, each dimension includes three entries. The scale was scored on a 5‐point scale, with 1 representing “*not at all*” and 5 representing “*completely*.” The higher the score is, the higher is the subject's level of being moved. The Cronbach's alpha coefficient for the scale in this study was .853.

#### Gratitude

The Chinese version of the gratitude scale (GQ‐6), revised by Wei et al. (2011), is used to measure gratitude. This scale has a total of six items (e.g., “There are too many things in my life that I feel thankful for.”), including two reverse scoring items, namely, Item 3 (“When I look at the world, I don't see much to be thankful for”) and Item 6 (“It takes me a lot of time to think of someone or something to be thankful for”). This is a 6‐point scale, with subjects' responses ranging from 1 (*strongly disagree*) to 6 (*strongly agree*), with higher scores representing higher levels of gratitude. The Cronbach's alpha coefficient for the questionnaire in this study was .807.

#### Prosocial behavior

The prosocial tendency measure (PTM) proposed by Kou et al. (2007) is used in this study to measure prosocial behavior. The scale consists of 26 questions, measuring six dimensions of prosocial behavior, namely, overt, anonymous, altruistic, compliance, emotional, and urgency. Four items measure the overt dimension (e.g., “I will do my best to help others when someone is present”). Five items measure the anonymous dimension (e.g., “I prefer to donate money anonymously”). Four items measure the altruistic dimension (e.g., “I donate money and goods not because I can benefit from them”). Five items measure the compliance dimension (e.g., “I seldom refuse help when others ask me for help”). Five items measure the emotional dimension (e.g., “I feel very good when I can comfort someone who is in a bad mood”). Finally, three items measure the urgency dimension (e.g., “I tend to help people who are in real trouble and in need of urgent help”). The scale is scored on a 5‐point scale, with responses ranging from 1 (*not at all conforming)* to 5 (*fully conforming*). Higher scores indicate higher prosocial tendencies. The Cronbach's alpha coefficient for the scale in this study was .81.

#### Empathic concerns

The interpersonal reactivity indicator scale (IRI‐C), revised by Zhang et al. (2010), is used in this study to measure empathic concerns. This scale consists of 22 items assessing four factors, namely, perspective‐taking, empathic concern, personal distress, and imagination. This study focuses more on the empathic concern dimension (e.g., “I often feel soft and caring towards those less fortunate than me”). Therefore, only that dimension is extracted from the full scale. The empathic concern subscale consists of six entries on a 5‐point scale, with subjects' responses ranging from 1 (*not at all*) to 5 (*completely*). Higher total scores indicate higher levels of empathy. The Cronbach's alpha coefficient for the empathic concern subscale in this study was .873.

### Data analysis

First, using the corrected item‐total correlations (Nunnally & Bernstein, 1994), an item analysis was performed on the Chinese GSS. Second, the SPSS19.0 software was used to conduct an EFA. The factor structure for the Chinese GSS items was determined via a maximum likelihood factor analysis with a Promax rotation. Then, the CFA via Mplus 8.3 was done to ensure that the measured indicators represented three latent factors. Values of 0.90 or higher for the comparative fit index (CFI) and Tucker–Lewis index (TLI) and a root‐mean‐square error of approximation (RMSEA) of 0.08 or lower serve as estimates of adequate fit (Hu & Bentler, 1995).

In addition, convergent, discriminant, and criterion validity have also been applied. The convergent validity coefficient and average variance extracted (AVE) are the indices used to test convergent validity. In this study, the latent variable's AVE had to be 0.50 or greater. Discriminant validity has been used to investigate the correlation between the three factors (emotion labels, tears of joy, warm feelings in the chest). The square roots of AVE were also used to compare the correlation coefficients of the three factors. If the correlation coefficient was smaller than all the square roots of AVE, then the discriminant validity was adequate (Hulland, 1999). The gratitude, empathic concerns, and prosocial behavior scale were used to test the criterion validity of the Chinese GSS. According to Cohen (1988), correlation coefficients < .30 suggest weak correlation; .30 to .50 suggest moderate correlation, and > .50 suggest strong correlation.

The internal consistency reliability was evaluated by Cronbach's *α*, composite reliability (CR), and retest coefficients. CR is regarded as the best internal consistency reliability estimate (Raykov & Grayson, 2003); A CR value of greater than 0.70 is acceptable (Hair et al., 1998). According to Barker et al. (1995), a Cronbach's *α* of .80–.89 suggests good, and a Cronbach's of ≥ .90 or higher suggests excellent reliability.

Finally, the measurement invariance across gender was determined by Mplus 8.3. This study separately confirmed the overall model fit of the CFA model for each gender. Then, the configural invariance was examined, followed by metric, scalar, and strict invariance. The *χ* ^2^ test is known to be oversensitive in the assessment of invariance in large samples (*N* > 300) (Chen, 2007). For that reason, the changes in CFI and RMSEA between the comparison and nested models were used for this assessment. Following Cheung and Rensvold (2002) and Chen (2007), ΔCFI < 0.010 and ΔRMSEA < 0.015 indicate a change in the fit that is not practically significant.

## RESULTS

### Item analysis

The data from Sample 1A were analyzed for item characteristics, and the scale scores were ranked from highest to lowest. Twenty‐seven percent of the highest and lowest scores were used as the criterion for high–low grouping; an independent samples *t*‐test was also performed for these groups (Nunnally & Bernstein, 1994). The results, shown in Table 1, indicate that each item achieved a statistically significant level of discriminatory power (*p* < .001). The correlation coefficients between each item's score and the total score of the corresponding dimension ranged from .69 to .86, with all total correlation coefficients meeting the criteria (*p <* .001).

**TABLE 1:** Item analysis of the Chinese GSS.

Item content	*T*	*r*
1. I often feel moved.	22.67***	.75***
2. I often feel touched.	19.01***	.75***
3. I am rarely moved.	15.94***	.59***
4. I often shed tears of joy.	17.19***	.63***
5. I often cry while having a warm feeling in the heart because I find something beautiful.	21.69***	.78***
6. I often feel a lump in my throat and get tears in my eyes, even though I am not sad.	15.07***	.56***
7. In the last month, there were many occasions in which I wanted to cry because I felt moved.	21.73***	.69***
8. It often warms my heart when people tell me touching stories.	17.28***	.72***
9. Listening to a moving story often gives me a warm sensation in the chest.	16.07***	.73***
10. Witnessing or hearing about positive stories often makes me experience a warm feeling in the heart.	14.45***	.68***

Abbreviation: GSS, Geneva Sentimentality Scale.

*** *p* < .001.

### Exploratory factor analysis

Using principal component analysis and promax oblique rotation, an EFA was performed on Sample 1A. The results show that the Kaiser–Meyer–Olkin value was 0.87, and the Bartlett's test of sphericity value was 72.2 (*p* < .001), indicating that the sample was suitable for EFA. To affirm cross‐cultural stability in the concept of being moved, the original three‐factor structure of the GSS was retained, fixing the factors to three for the analysis to test the scale's applicability in a Chinese context. The results show that the first factor accounted for 27.36% of the variance, and the second factor explained 49.53% of the variance. The total variance explained was 71.24%. However, after rotation, the distribution of the factors was uneven. The fifth item had factor loadings on both Factor 1 and Factor 2, with loadings below 0.5 for both factors, and therefore, the fifth item was deleted. This increased the total explained variance to 72.92% and resulted in an even distribution of each factor among the dimensions. As shown in Table 2, the factor loadings of the remaining items ranged from 0.71 to 0.88. Through this process, the inappropriate item (Question 5) was removed, resulting in a final Chinese GSS with nine items and three factors.

**TABLE 2:** Exploratory factor analysis results.

Item content	Factor loading		
	3	2	1
1. I often feel moved.	‐	‐	0.786
2. I often feel touched.	‐	‐	0.718
3. I am rarely moved.	‐	‐	0.797
4. I often shed tears of joy.	‐	0.738	‐
6. I often feel a lump in my throat and get tears in my eyes, even though I am not sad.	‐	0.822	‐
7. In the last month, there were many occasions in which I wanted to cry because I felt moved.	‐	0.749	‐
8. It often warms my heart when people tell me touching stories.	0.838	‐	‐
9. Listening to a moving story often gives me a warm sensation in the chest.	0.883	‐	‐
10. Witnessing or hearing about positive stories often makes me experience a warm feeling in the heart.	0.862	‐	‐
% of variance explained	72.916		

In summary, the item and EFA analyses show that the Chinese GSS has good construct validity.

#### Confirmatory factor analysis

**FIGURE 1:**
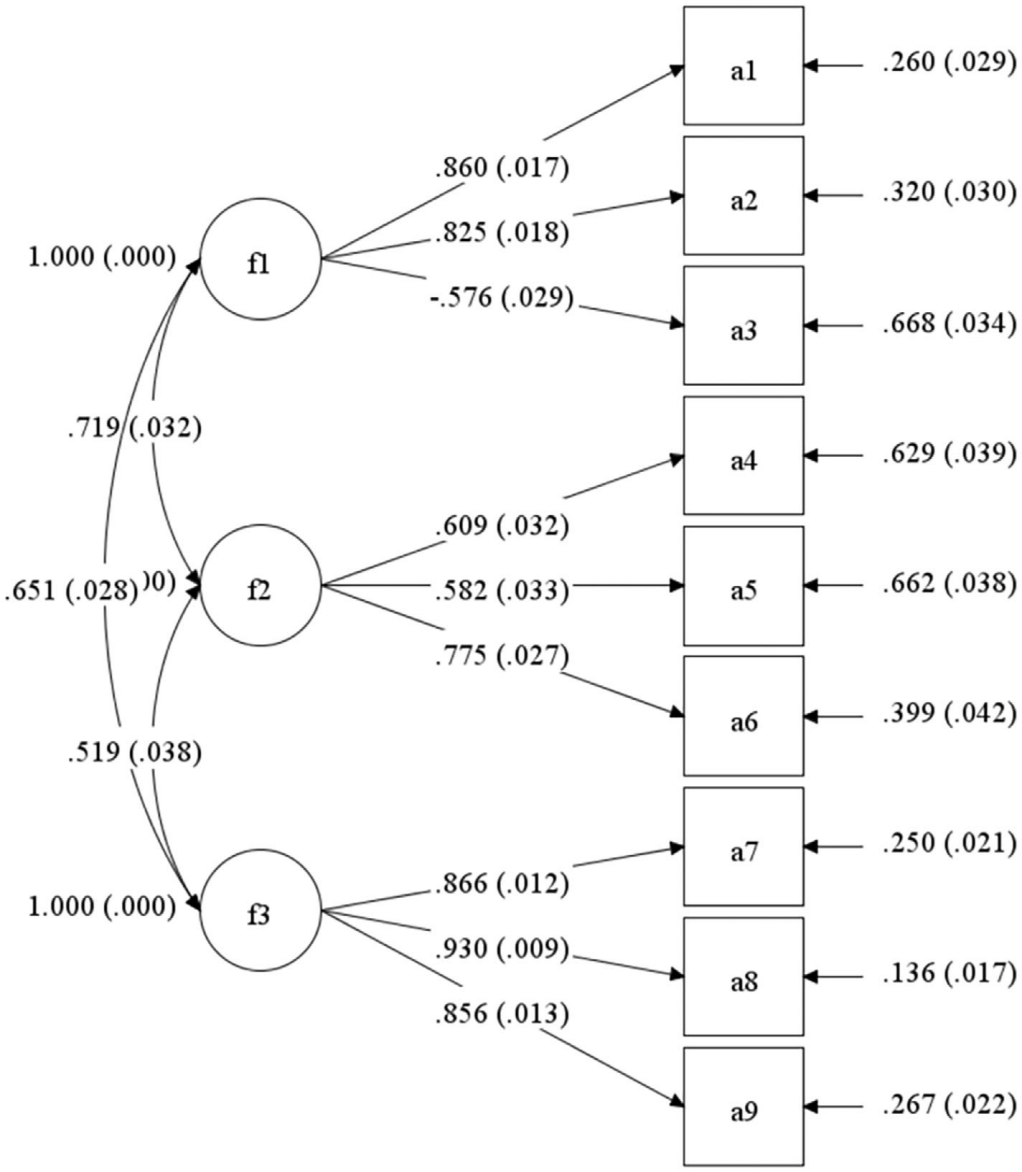
Confirmatory factory analysis model with standardized regression weights. f1 = Emotion labels; f2 = Tears of joy; f3 = Warm feelings in the chest.

A CFA was conducted on Sample 1B (see Figure 1). Because the chi‐squared statistic is dependent on sample size, a trivial difference can lead to a significant chi‐squared statistic in large samples (Rigdon, 1995). Thus, this study focused on the other fit indices. The results indicate that the three‐factor GSS model was obviously the model with the best fit (*χ* ^2^[24] = 68.554, *p <* .001; CFI = 0.985; TLI = 0.977; RMSEA = 0.053, 90% CI [0.039, 0.068]).

#### Convergent and discriminant validity

The AVE values of each factor of the GSS were 0.584, 0.437, and 0.783, all of which were greater than 0.04. The values were 0.804, 0.696, and 0.915, which indicate that the convergent validity of the scale was at an acceptable level. As shown in Table 3, all three factors are significantly correlated with each other, and none of their correlations exceed the square root of their corresponding AVEs. This all suggests that the Chinese GSS has good discriminant validity.

**TABLE 3:** Discriminant validity of the Chinese GSS.

	Emotion labels	Tears of joy	Warm feelings in the chest
Emotion labels	1		
Tears of joy	0.428***	1	
Warm feelings in the chest	0.476***	0.297***	1
The square roots of AVE	0.764	0.661	0.885

Abbreviations: AVE, average variance extracted; GSS, Geneva Sentimentality Scale.

*** *p* < .001.

#### Criterion validity analysis

This study also calculated the bivariate Pearson's correlation coefficients of gratitude, empathic concerns, the prosocial behavior scores, and the Chinese GSS score. The results show that being moved was positively associated with gratitude (*r* = .408, *p <* .001), empathic concerns (*r =* .276, *p <* .001), and prosocial behavior (*r =* .394, *p < .0*01).

#### Measurement invariance across gender

In recent years, extensive academic attention has been devoted to discovering whether instruments have measurement equivalence in terms of gender. Therefore, this study uses validation factor analysis to test the cross‐gender equivalence of the Chinese GSS. First, to obtain a single‐group baseline model, the total male and female samples obtained from this survey were subjected to validation factor analysis. The results show that each baseline model met the psychometric acceptance criteria.

Then, the Kolmogorov‐Smirnov normality test was conducted on the data of each item of the Chinese GSS. The results show that the data of each item were nonnormally distributed (*p <* .001). It was therefore necessary to use robust great likelihood estimation to conduct multi‐group validation analyses. The four models were established for the Chinese GSS, and the results, shown in Table 4, indicate that the fitting indices of each model are better. A step‐by‐step comparison of the models reveals that the BIC value decreases gradually. As shown in Table 4, the results indicate that the Chinese GSS is not affected by gender. To be more specific, the configural invariance model fits the data very well (RMSEA = 0.039 [90% CI, 0.017–0.056], CFI = 0.987). A constrained metric invariance model showed an acceptable fit (RMSEA = 0.034 [90% CI, 0.010–0.052], CFI = 0.989), and the model (M2) fits the data very well (RMSEA = 0.035 [90% CI, 0.015–0.052], CFI = 0.986). In addition, a more rigorous modelling (M3) was performed. As shown in Table 4, the ΔCFI was −0.029, and ΔRMSEA was 0.023. Thus, it is clear that the Chinese GSS has scalar but not strict invariance across gender.

**TABLE 4:** Tests of measurement invariance across genders.

Model	*χ* ^2^	Df	TLI	CFI	RMSEA (90%CI)	SRMR	BIC	ΔCFI	ΔRMSEA
Gender	‐	‐	‐	‐	‐	‐	‐	‐	‐
Male (*n* = 214)	35.28	24	0.982	0.988	0.047 (0.000–0.078)	0.037	‐	‐	‐
Female (*n* = 443)	52.274	24	0.977	0.985	0.052 (0.032–0.071)	0.033	‐	‐	‐
M0: Configural invariance	71.439	48	0.981	0.987	0.039 (0.017–0.056)	0.034	14,161.432	‐	‐
M1: Metric invariance	74.34	54	0.985	0.989	0.034 (0.010–0.052)	0.04	14,128.352	0.002	−0.005
M2: Scalar invariance	84.709	60	0.984	0.986	0.035 (0.015–0.052)	0.044	14,100.463	−0.003	0.001
M3: Strict invariance	146.001	69	0.956	0.957	0.058 (0.045–0.071)	0.067	14,130.637	−0.029	0.023

Abbreviations: BIC, Bayesian information criterion; CFI, comparative fit index; RMSEA, root‐mean‐square error of approximation; SRMR, standardized root‐mean square residual; TLI, Tucker–Lewis index.

#### Reliability analysis

Cronbach's *α*, CR, and retest coefficients were selected as the internal consistency reliability indicators in this study to test the 1328 valid questionnaires in Sample 1. The Cronbach's *α* for the Chinese GSS scale was.853, with the three dimensions of emotion labels, tears of joy, and warm feelings in the chest scoring .788, .711, and .900, respectively. However, Cronbach's *α* has been criticized as a lower bound that frequently underestimates true reliability. Therefore, this study estimated CR, a popular alternative. The CR coefficients for emotion labels, tears of joy, and warm feelings in the chest were .805, .714, and .903, respectively, and the overall Chinese GSS score was 0.833. Finally, Pearson's *r* correlations were used to determine the stability over time of the Chinese GSS. The total score remained stable over an 8‐week interval (*r* = .712, *p* < .001), and all indicators demonstrated that the Chinese GSS had acceptable reliability.

## DISCUSSION

Given the importance and reliability of the GSS in existing research literature, this study tested the GSS with college students in a Chinese cultural context. The results of the analysis showed that the Chinese GSS items were significantly correlated with the total scores of their respective dimensions. In addition, the items differed significantly between high and low groups. Therefore, the Chinese GSS demonstrates good differentiation ability. Second, the reliability analysis showed that the Cronbach's *α* coefficient of the scale was .853, indicating strong internal consistency and stability. Third, the results of the EFA show that, after deleting the fifth item, the distribution of each item across factors was uniform. In addition, the overall interpretability increased. CFA also revealed that, after the deletion of the fifth item, the three‐factor model was well‐fitted. Thus, the Chinese version of the three‐factor nine‐item GSS has good structural validity. These results indicate that the GSS has high validity and reliability in the Chinese cultural context. This confirms that the GSS is a valid research tool that can be used for assessing the degree of “being moved” in people.

The inapplicability of the fifth item of the GSS in a Chinese cultural context can be attributed to several cultural factors. First, emotional expression is closely tied to cultural norms, which in turn influence and shape individual emotional behaviors. Under the influence of Confucianism and the value of mediocrity, emotional expressions among Chinese people are typically more introverted and context‐dependent (Zhou & Wang, 2012). In this study, both the text and the online format of the questionnaire conveyed to the participants a formal and rational emotional atmosphere. Despite the instructions emphasizing that participants should “answer in accordance with your true feelings,” they tended to express their emotions in a rational manner, one that is more respected in Chinese culture. This cultural tendency can inhibit high emotional arousal behaviors, such as shedding tears. Furthermore, Yang (2009) posited that the middle‐of‐the‐road thinking—which is prevalent in Chinese culture—affects individuals' emotional expressions and arousal. Emotional expression aimed at fostering “internal and external harmony” and facilitating interpersonal communication is not seen as negative repression but rather as positive reinforcement of social conduct. Therefore, as they manage their emotions, suppressing tears in formal situations is a common behavioral norm for Chinese individuals. In terms of emotional arousal, individuals influenced by middle‐of‐the‐road thinking focus on rational judgment before they allow themselves to experience emotional responses. They consider the environment and relevant events to avoid large fluctuations in their emotions and to maintain harmony. As a result, intense emotional expressions such as tears (which indicate high emotional arousal) are difficult for Chinese people to express under the dual constraints of rationality and harmony. Consequently, the scale's fifth item, “shedding tears upon discovering beauty,” which denotes an overt emotional display, is less applicable to Chinese individuals. This is especially true in a formal study setting, which reduces the measurement validity of this item within the Chinese cultural context.

In terms of the scale's calibration validity, the results of this study demonstrate that the Chinese GSS possesses good validity. In this study, being moved was significantly and positively correlated with gratitude, prosocial behavioral tendencies, and empathic concerns, findings aligning with those of existing studies. First, gratitude refers to the psychological tendency of individuals to respond with appreciative cognition, emotion, and behavior to positive experiences or outcomes resulting from another person's favor or help (Wei et al., 2011). According to the theory of positive core values proposed by Cova and Deonna (2014), being moved evokes an individual's positive core values. Consequently, when individuals perceive that they have received a favor from another person, they are more likely to reciprocate under the influence of these positive core values. This reciprocal behavior is a physical manifestation of gratitude. Second, in terms of prosocial behavioral tendencies, researchers have found that being moved can stimulate interpersonal caring and empathy (Fiske et al., 2019). Thus, being moved can promote individuals' commitment to their relationships. This commitment can further increase prosocial behavioral tendencies (Seibt et al., 2017), such as offering help to others. Such an emotion not only influences interpersonal interactions but can also extend to group relationships, promoting intergroup social identity, mitigating emotional polarization, and increasing prosocial behavior between groups. Finally, extensive research has indicated the existence of a strong correlation between being moved and empathy. For example, Zickfeld, Schubert, Seibt, Blomster, et al. (2019), Zickfeld, Schubert, Seibt, and Fiske (2019) found that empathic concern is a personality trait that predicts the frequency and intensity of an individual's experience of kama muta. This finding in turn suggests that empathic concern may be a susceptibility factor for kama muta. The cross‐cultural study demonstrated that the high correlation between being moved and empathic concern remains stable across cultural differences. Similarly, Steinnes et al. (2019) confirmed that the trait of kama muta is highly correlated with empathic concern. Building on previous research, the results of this study provide robust evidence of the inextricable relationship between being moved and empathy.

The debate between rationality and emotionality has a long history, particularly in discussions of gender issues. Given that being moved is typically associated with sensitivity, this study conducted a cross‐gender measurement equivalence test for the GSS. The specific aim was to investigate potential measurement differences between genders. The results show, however, that the scale has equivalent measurement significance for between‐group comparisons. To some extent, this finding supports the view that there are no gender differences in affective responses. Kelly et al. (2008) assessed several physiological (e.g., cortisol reactivity and heart rate) and psychological (e.g., depression, irritability, anger, and fear) metrics in male and female subjects using the Trier social stress test (TSST). The study found that, following the TSST, while women reported more fear, irritability, confusion, and less well‐being, there were no significant differences in cortisol reactivity between genders. Furthermore, the degree of autonomic response did not show significant male–female differences. The overall indication was that there are no significant differences in the physiological expression of emotional responses between men and women. This lack of difference aligns with evolutionary psychology perspectives. For instance, researchers studying cuteness (Steinnes et al., 2019) have found that cuteness significantly evokes kama muta. In daily life, people may experience similar emotional responses when seeing cute babies, including watery eyes, a warm feeling, and goosebumps, all of which can motivate caregiving behaviors. During human evolution, the emotion of being moved and the resulting behavioral responses have played a crucial role in ensuring both survival and reproduction. This “being moved” emotion is theorized to have evolved with adaptive significance, becoming an inherent response shared universally across humanity. Thus, regardless of race and gender, this emotion is stable in humans, with the only differences being the degree and type of stimuli experienced.

## CONTRIBUTION AND LIMITATIONS

The results of this study offer valuable insights for future research. Firstly, this GSS tool can be used to explore the frequency of being moved and the corresponding physiological responses within the context of Chinese culture, effectively promoting the study of this unique emotion in China. Secondly, the results of this study confirm the cross‐cultural consistency of responses to being moved, which in turn suggests that the physiological responses to being moved are highly consistent across different cultural backgrounds. This indicates that, despite the complexity of factors that elicit being moved, this emotion may be one of the most basic of those evolved in humans, similar to happiness and anger. Subsequent research will build on this premise to clarify the impact of the “being moved” emotion on human and social development.

Despite the meaningful findings in this study, there are still some limitations. Firstly, all data indicators show that the GSS is suitable for use in the Chinese cultural context and ensures sufficient reliability and validity. However, the study only included young college students as research subjects. Future research should expand the scope of subjects to include junior and senior high school students, working professionals, and others. Expanding the sample would help to more accurately assess whether the GSS maintains its measurement efficacy. Secondly, the results of this study suggest that the components of being moved (e.g., tears, inner warmth) measured by the GSS are somewhat culturally universal. However, given the profound cultural disparities between China and the West, a question arises: Are the elements and constructs of being moved distinctive within the Chinese cultural context? This question requires further research, such as in‐depth interview analyses, to further elucidate the components of being moved in Chinese culture.

## CONCLUSION

In conclusion, the Chinese GSS exhibits strong psychometric characteristics, making this scale a reliable measurement tool in Chinese settings. This result demonstrates that the GSS maintains its reliability across different cultural contexts and holds significant implications for future cross‐cultural research.

## CONFLICT OF INTEREST STATEMENT

The authors declare no conflicts of interest.

## ETHICS STATEMENT

This study had been reviewed by the Ethics Committee of Southwest University (IRB NO. H24090) and had been carried out in accordance with the provisions of the Declaration of Helsinki. All subjects informed the content and objectives of this study, and then the participant signed the consent form.
